# Plasmodesmata-Dependent Intercellular Movement of Bacterial Effectors

**DOI:** 10.3389/fpls.2021.640277

**Published:** 2021-03-22

**Authors:** Zhongpeng Li, Haris Variz, Yani Chen, Su-Ling Liu, Kyaw Aung

**Affiliations:** Department of Genetics, Development, and Cell Biology, Iowa State University, Ames, IA, United States

**Keywords:** *Pseudomonas syringae*, plasmodesmata-located protein, flg22, callose, *Nicotiana benthamiana*

## Abstract

Pathogenic microorganisms deliver protein effectors into host cells to suppress host immune responses. Recent findings reveal that phytopathogens manipulate the function of plant cell-to-cell communication channels known as plasmodesmata (PD) to promote diseases. Several bacterial and filamentous pathogen effectors have been shown to regulate PD in their host cells. A few effectors of filamentous pathogens have been reported to move from the infected cells to neighboring plant cells through PD; however, it is unclear whether bacterial effectors can traffic through PD in plants. In this study, we determined the intercellular movement of *Pseudomonas syringae* pv. *tomato* (*Pst*) DC3000 effectors between adjoining plant cells in *Nicotiana benthamiana*. We observed that at least 16 *Pst* DC3000 effectors have the capacity to move from transformed cells to the surrounding plant cells. The movement of the effectors is largely dependent on their molecular weights. The expression of PD regulators, *Arabidopsis* PD-located protein PDLP5 and PDLP7, leads to PD closure and inhibits the PD-dependent movement of a bacterial effector in *N. benthamiana*. Similarly, a 22-amino acid peptide of bacterial flagellin (flg22) treatment induces PD closure and suppresses the movement of a bacterial effector in *N. benthamiana*. Among the mobile effectors, HopAF1 and HopA1 are localized to the plasma membrane (PM) in plant cells. Interestingly, the PM association of HopAF1 does not negatively affect the PD-dependent movement. Together, our findings demonstrate that bacterial effectors are able to move intercellularly through PD in plants.

## Introduction

Plasmodesmata (PD) are membrane-lined channels which physically connect adjoining plant cells. PD provide the symplastic pathway for the connected cells to exchange molecules directly (Lucas et al., 2009; Brunkard and Zambryski, 2017; Nicolas et al., 2017). The PD-dependent movement of hormones, sugars, proteins, and RNAs has been well documented (Kragler, 2013; Schulz, 2015; Kitagawa and Jackson, 2017; Reagan et al., 2018). In addition to their fundamental roles in plant growth and development, recent findings highlighted the crucial roles of PD in plant immunity (Cheval and Faulkner, 2018).

Plasmodesmata enable the continuity of the plasma membrane (PM) and endoplasmic reticulum (ER) and link the cytoplasm of adjoining plant cells. The space between the PM and ER membrane lining, known as cytoplasmic sleeve, allows the trafficking of molecules between the adjoining plant cells. The function of PD is largely defined by their aperture in permitting molecules to move across. The largest molecules that can traffic through the cytoplasmic sleeve are known as the size exclusion limit (SEL; Kim and Zambryski, 2005). Soluble green fluorescent proteins (1×sGFP; 27 kDa) can freely move between adjoining plant cells through PD, whereas the movement of 2×sGFP (54 kDa) and 3×sGFP (71 kDa) is largely inhibited between physically connected cells in *Arabidopsis* (Kim et al., 2005; Aung et al., 2020). Among different regulators, callose plays the most prominent role in regulating the PD function. Callose is a plant polysaccharide, which is deposited in the cell wall around the PM lining of PD. The accumulation and degradation of callose at PD allow plant cells to dynamically control the closing and opening of PD. Callose deposition at PD is positively correlated with PD closure. Callose synthase (CalS) and β-1,3-glucanase are involved in callose biosynthesis and degradation, respectively (De Storme and Geelen, 2014; Wu et al., 2018).

In addition to the enzymes directly involved in regulating callose homeosis at PD, plasmodesmata-located proteins (PDLPs) play critical roles in modulating the plasmodesmal function. PDLPs affect callose homeostasis at PD by an unknown mechanism. Ectopic expression of PDLP5 results in overaccumulation of callose, whereas a *pdlp5* knock-out mutant accumulates much less callose at PD compared to that of wild type in *Arabidopsis* (Lee et al., 2011). The expression of *PDLP5* transcripts is upregulated by *Pseudomonas syringae* pv. *maculicola* ES4326 infection (Lee et al., 2008, 2011). PDLP1 accumulates at the PM and haustorial interfaces during *Hyaloperonospora arabidopsidis* (*Hpa*) infection (Caillaud et al., 2014). The polar localization of PDLP1 at the haustorium leads to callose deposition at the interface (Caillaud et al., 2014). Despite the involvement of PDLP1 during *Hpa* infection, it is yet to establish whether the plasmodesmal immunity is involved. It has also been demonstrated that pathogen-associated molecular patterns (PAMPs), the fungal cell wall PAMP (chitin) or a 22-amino acid peptide of bacterial flagellin (flg22), are sufficient to trigger callose deposition at PD in *Arabidopsis* (Faulkner et al., 2013; Xu et al., 2017).

Recent findings began to reveal that pathogenic microbes utilize protein effectors to modulate the PD function in their hosts. Microbial effectors are known for altering plant cellular processes to suppress plant immunity (Cui et al., 2015; Buttner, 2016). The fungal pathogen *Fusarium oxysporum* effectors Avr2 and Six5 are localized to PD when transiently overexpressed in *Nicotiana benthamiana* (Cao et al., 2018). The two PD-localized effectors form heterodimer and regulate the plasmodesmal function to allow larger molecules to traffic through PD. The oomycete pathogen *Phytophthora brassicae* RxLR3 effector is localized to PD and physically associated with CalSs, CalS1, CalS2, and CalS3, when transiently overexpressed in *N. benthamiana*. RxLR3 expressed in *Arabidopsis* transgenic plants suppresses the function of the CalSs, inhibiting the callose accumulation at PD. In addition, transient overexpression of RxLR3 in *N. benthamiana* promotes the PD-dependent movement of fluorescent molecules between cells (Tomczynska et al., 2020). The bacterial pathogen *Pseudomonas syringae* pv. *tomato* DC3000 (*Pst* DC3000) delivers effector HopO1-1 to regulate the PD function. HopO1-1 expressed in *Arabidopsis* transgenic plants degrades PDLP5-YFP and PDLP7-YFP. In addition, *Pst* DC3000 promotes the degradation of PDLP7-HF in *Arabidopsis* in a HopO1-1-dependent manner during the infection (Aung et al., 2020). Together, the reports showed that pathogenic microbes use effectors to target different PD regulators.

*Pseudomonas syringae* pv. *tomato* DC3000 deploys 36 effectors into host cells through type III secretion system (Lindeberg et al., 2012; Wei et al., 2015). The regulation of PD by HopO1-1 prompted us to investigate whether *Pst* DC3000 effectors can move through PD. We determined the PD-dependent movements of *Pst* DC3000 effectors. We also explored whether PDLP5, PDLP7, and flg22 affect the PD-dependent movement of bacterial effectors between plant cells.

## Materials and Methods

### Plant Growth Conditions

*Nicotiana benthamiana* plants were grown at 22°C with 50% humidity and irradiated with 120 μmol m^−2^ s^−1^ white light for 14 h per day.

### Gene Cloning and Plasmid Construction

To generate effector fused to two tandem repeats of yellow fluorescent protein (YFP), the coding sequence of effectors and YFP without a stop codon was amplified from effector-YFP (Aung et al., 2020) and pGW2-YFP (Reumann et al., 2009), respectively. PCR products of effectors and YFP were fused together using an overlapping PCR method with Gateway-compatible primers as described previously (Aung et al., 2020). The stitched PCR fragments were cloned into pDONR 207 and a destination vector pGW2-YFP (Reumann et al., 2009) using a standard Gateway cloning system (Invitrogen). To construct HopAF1^G2A^-YFP, the coding sequencing of HopAF1^G2A^ was amplified from HopAF1-YFP using Gateway-compatible primers. A G to A mutation was introduced in the forward primer. The PCR product was cloned into pDONR 207 and then pGW2-YFP (Reumann et al., 2009). To construct a HF-mCherry construct, the coding sequence of mCherry was amplified from mCherry-pTA7002 (Fujioka et al., 2007) using Gateway-compatible primers. The PCR product was cloned into pDONR 207 and then pB7-HFN-stop (Lee et al., 2017). All primers used for cloning are listed in Supplementary Table 1.

### Agrobacterium-Mediated Transient Expression for Subcellular Localization, Immunoblot Analysis, and PD-Dependent Movement Assay

*Agrobacterium tumefaciens* GV3101 harboring different expression constructs were cultured in a 30°C shaking incubator overnight. The overnight cultures were adjusted to a desired bacterial density using sterilized ddH_2_O. The bacterial solutions were infiltrated into the fourth leaves of 5-week-old *N. benthamiana* plants. For subcellular localization analysis, and immunoblot analysis a bacterial culture with an optical density of 0.1 (A_600_) was used. *Nicotiana benthamiana* leaves were collected 2-days after infiltration for confocal imaging or immunoblot assays.

To establish an Agrobacterium-mediated protein movement assay, we followed the method previously described (Brunkard et al., 2015). *35S::His-Flag (HF)-YFP* or *35S::HopAF1-YFP* (Aung et al., 2020) was transformed into Agrobacterium harboring *35S::ER-CFP* (Nelson et al., 2007). The resulting Agrobacterium carrying two different plasmid DNAs were infiltrated into the fourth leaves of 5-week-old *N. benthamiana* at an optical density of 2 × 10^−4^ (A_600_). The expression of the fusion proteins was detected 2 days post infection using confocal microscopy as described below. Plant cells expressing ER-CFP were designated as the transformed plant cells. The movement of HF-YFP or HopAF1-YFP was determined by the detection of YFP signals surrounding the transformed cells. About 76 and 52 images were captured from three biological replicates for HF-YFP and HopAF1-YFP, respectively. An independent Agrobacterium infiltration into different *N. benthamiana* plants was defined as a biological replicate. If YFP was only detected in the transformed cells, they were scored as 0. If YFP was detected in cells physically connecting the transformed cells, they were scored as 1. If YFP diffused beyond the first cell layer from the transformed cells, they were scored as ≥2. To compare the movement between YFP molecules, the numbers of surrounding plant cells to the transformed cells containing YFP signals were counted and analyzed.

To determine the SEL of *N. benthamiana*, the movement of 1×YFP, 2×YFP, and 3×YFP (Aung et al., 2020) was investigated as mentioned above. About 71, 94, and 81 images were captured from three biological replicates for 1×YFP, 2×YFP, and 3×YFP, respectively.

To determine the movement of effectors between plant cells, bacterial effector-YFP fusion proteins (Aung et al., 2020) were transiently expressed in *N. benthamiana* and examined as mentioned above. Plant cells with the strongest YFP signals were designated as the transformed plant cells. The movement of the effector fusion proteins was determined by the detection of YFP signals surrounding the transformed cells. More than 100 transformation events were imaged across at least three biological replicates for all effectors except AvrE-YFP. Around 48 images were collected for AvrE-YFP from three biological replicates.

To determine the effect of PDLP5 and PDLP7 on the movement of the bacterial effector HopAF1, the fourth leaves of 5-week-old *N. benthamiana* plants were infiltrated with mixtures of Agrobacterium harboring *35S::PDLP5-HF* (A_600_ 0.1), *35S::PDLP7-HF* (A_600_ 0.1), or *35S::HF-mCherry* (A_600_ 0.1) with *35S::HopAF1-YFP* (A_600_ 2 × 10^−4^). The movement of HopAF1-YFP was determined by the detection of YFP signals surrounding the transformed cells as described above. The numbers of surrounding plant cells to the transformed cells containing YFP signals were counted and compared. More than 100 images collected from three biological replicates were analyzed.

To determine the effect of flg22 on the movement of a bacterial effector HopAF1, 0.1 μM of flg22 was infiltrated into fully expanded leaves of 5-week-old *N. benthamiana*. For a mock treatment, ddH_2_0 was infiltrated. Twenty-four hours after the treatment, Agrobacteria harboring *35S::HopAF1-YFP* were infiltrated into the mock‐ or flg22-treated leaves. More than 100 images collected from three biological replicates were analyzed.

### Plasmodesmal Callose Staining Assay

The fourth leaf of *N. benthamiana* was infiltrated with ddH_2_O (mock) or 0.1 μM of flg22 for 24 h. Aniline blue (0.01% in 1×PBS buffer, pH 7.4) was infiltrated into the treated area to image callose accumulation at PD as previously described (Xu et al., 2017). To determine the role of PDLP5 and PDLP7 in callose accumulation, Agrobacteria harboring *35S::PDLP5-HF*, *35S::PDLP7-HF*, and *35S::HF-YFP* (mock) were infiltrated into the fourth leaf of *N. benthamiana*. Aniline blue was infiltrated into the bacterial infected area 48 h post infection for imaging callose accumulation at PD as mentioned above. Plasmodesmal callose deposits were imaged using confocal microscopy 15 min after dye infiltration. Around 10 images were collected from each sample. Aniline blue stained callose was quantified using the Macro feature of FIJI for large scale data analysis. In brief, images were first converted from lsm to tif and then to eight-bit image files. RenyiEntropy white method was used to set Auto Threshold creating black and white images highlighting callose. Particle Analysis tool was used to outline each aniline blue-stained callose and ascribe a quantitative numerical value in μm^2^. Exclusion setting of 0.10–20 μm^2^ and a circularity of 0.30–1.00 were used to isolate callose excluding any non callose related fluorescence. About 10 images were collected from each treatment. Data from 10 images from an experiment were pooled and plotted as mentioned below.

### FM4-64 staining

Around 50 μM of FM4-64 dye (Life Technologies) was infiltrated into the bacterial infected area of *N. benthamiana* leaves 48 h post infection for staining the PM. HopAF1-YFP, HopAF1^G2A^-YFP, and FM4-64 signals were imaged using confocal microscopy 3 h after the dye infiltration.

### Confocal Imaging

Zeiss Laser Scanning Microscopy 700 was used to image fluorescent signals. For subcellular localization and imaging aniline blue-stained callose, a small piece (~4 mm^2^) of leaf tissues was mounted with water on a glass slide with the abaxial side facing upward. For imaging PD-dependent movement of fluorescent molecules, a larger piece (~1 cm^2^) of leaf tissues was mounted with water on a glass slide with the abaxial side facing upward. Different fluorescent signals were excited with the following laser lines: callose (405 nm), YFP (488 nm), CFP (405 nm), and FM4-64 (555 nm). The signals were then collected using the following emission filters: callose (SP 555), YFP (SP 555), CFP (SP 555), and FM4-64 (SP 640).

### Statistical Analysis

All presented experiments were performed at least three independent times. The pooling of data from different biological replicates for different experiments is indicated in each section. Violin box plots were created with an online software.^1^

Mann-Whitney *U* Test^2^ was performed for testing statistical significance of differences.

### Immunoblot Analyses

*N. benthamiana* leaves were frozen with liquid nitrogen and homogenized with 1600 miniG (SPEX). Protein extraction buffer [60 mM Tris-HCl (pH 8.8), 2% (v/v) glycerol, 0.13 mM EDTA (pH 8.0), and 1× protease inhibitor cocktail complete from Roche] was added to the homogenized tissues (100 μl/10 mg). The samples were vortexed for 30 s, heated at 70°C for 10 min, and centrifuged at 13,000 *g* for 5 min at room temperature. The supernatants were then transferred to new tubes. For SDS-PAGE analysis, 10 μl of the extract in 1x Laemmli sample buffer (Bio-Rad) was separated on 4–15% Mini-PROTEAN TGX precast protein gel (Bio-Rad). The separated proteins were transferred to a polyvinylidene fluoride membrane (Bio-Rad) using a Trans-Blot Turbo Transfer System RTA transfer kit following the manufacturer’s instructions (Bio-Rad). The membrane was incubated in a blocking buffer [3% (v/v) BSA, 50 mM Tris base, 150 mM NaCl, 0.05% (v/v) Tween 20 (pH 8.0)] at room temperature for 1 h, then incubated overnight with an antibody prepared in the blocking buffer at 4°C overnight. The antibodies used are as follows: 1:20,000 anti-GFP (Abcam catalog No. ab290), 1:10,000 anti-cMyc (Abcam catalog No. ab9106), and 1:10,000 anti-Flag-HRP (Sigma-Aldrich catalog No. A8592). The probed membranes were washed three times with 1× TBST [50 mM Tris base, 150 mM NaCl, and 0.05% (v/v) Tween 20, pH 8.0] for 5 min before being incubated with a secondary antibody at room temperature for 1 h except for anti-Flag-HRP. The secondary antibodies used were 1:20,000 goat anti-rabbit IgG (Thermo Fisher Scientific catalog No. 31,460). Finally, the membranes were washed four times with 1× TBST for 10 min before the signals were visualized with SuperSignal West Dura Extended Duration Substrate (Pierce Biotechnology).

## Results

### An Agrobacterium-Mediated Protein Movement Assay in *Nicotiana benthamiana*

The PD-dependent movement of fluorescent molecules has been previously established in *N. benthamiana* (Brunkard et al., 2015). To unambiguously locate Agrobacterium infected plant cells, we infiltrated Agrobacterium harboring two plasmids (*35S::ER-CFP and 35S::HF-YFP*) into *N. benthamiana* at an optical density of 2 × 10^−4^ (A_600_), resulting in a few transformed cells per cm^2^ of leaf surface. Free YFP molecules are able to move between cells through PD, whereas ER-CFP cannot move through PD from the transformed cells. Thus, the expression of ER-CFP can be used to locate the transformed plant cells. Using confocal microscopy, we observed transformation events on the epidermis of *N. benthamiana*, determined by the expression of ER-CFP. We imaged 76 transformation events from three biological repeats. We observed that the transformed plant cells always express the strongest YFP signals within a cluster of cells containing YFP. Plant cells containing weaker YFP signals surrounding the transformed cell are resulted from the PD-dependent movement of YFP (Supplementary Figure 1A). We thus concluded that the plant cells containing the strongest YFP signals could be used to identify the transformed plant cells using the experimental system. In addition, we tested the SEL of PD in *N. benthamiana* epidermis. It has been established that 1×YFP (~27 kDa) can effectively move through PD between *Arabidopsis* epidermal cells, whereas the movement of YFP concatemer 2×YFP (~54 kDa) was greatly inhibited. No movement of 3×YFP (~81 kDa) was observed in *Arabidopsis* (Aung et al., 2020). Using the Agrobacterium-mediated protein movement assay in *N. benthamiana*, we observed the PD-dependent movement of 1×YFP in all transformation events detected (Supplementary Figure 1B). Around 20% of the transformation events of 1×YFP led to the diffusion of two or more than two cell layers. 2×YFP and 3×YFP resulted in around 30 and 10% PD-dependent trafficking, respectively (Supplementary Figure 1C). More strikingly, 1×YFP diffused to an average of 5.5 cells, whereas the concatemers moved to less than one cell (Supplementary Figure 1C).

### Bacterial Effectors Traffic Between Plant Cells

**Figure 1:**
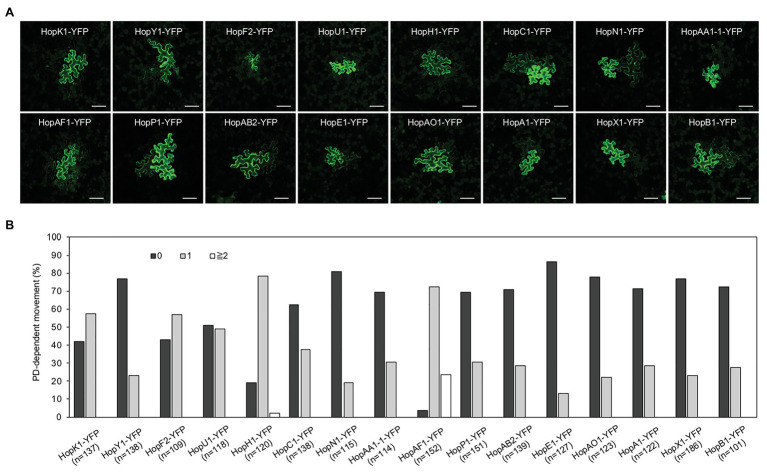
The movement of bacterial effectors between plant cells. **(A)** Confocal images show the diffusion of effector-yellow fluorescent protein (YFP) fusion proteins. Images were taken from epidermis of *Nicotiana benthamiana* leaves. An agrobacterium-mediated protein movement assay was conducted to determine the movement of effector-YFP fusion proteins in plants. The transformed plant cell exhibits strong yellow fluorescent (YFP) signals. The movement of the fusion proteins is determined by the detection of YFP signals in cells surrounding the transformed cell. Scale bars = 100 μm. **(B)** Quantitative data show the percentage of transformation events resulting in no diffusion (0), one cell layer diffusion (1), and two or more than 2 cell layers diffusion (≧2). The data shown here are pooled from at least three biological replicates. The number of transformation events analyzed is indicated (n).

To determine whether effectors can move from the infected cells to the surrounding plant cells, we monitored the movement of YFP tagged *Pst* DC3000 effector (Aung et al., 2020). 29 *Pst* DC3000 effector-YFPs were transiently expressed in *N. benthamiana*. Transient expression of some effectors led to cell death (Supplementary Table 2), whereas the expression of a few effectors could not be detected. Although the expression of some effectors using higher Agrobacterium inoculum (A_600_ 0.1) leads to cell death, lower Agrobacterium inoculum (A_600_ 2 × 10^−4^) allows us to detect the expression of the fusion proteins 2 days after infiltration. We selected 17 effectors to further investigate their movement between plant cells. Confocal images showed the expression of 16 effector-YFP fusion proteins in the epidermis *N. benthamiana* leaves (Supplementary Figure 2). It is noted that the expression of HopH1-YFP, HopN1-YFP, HopAO1-YFP, HopA1-YFP, and AvrE-YFP using a higher Agrobacterium inoculum (A_600_ 0.1) leads to cell death in *N. benthamiana*. Among the selected effectors, we observed the movement of 16 bacterial effectors between plant cells (Figure 1A). More than 50% of transformation events lead to the intercellular movement of HopK1-YFP, HopF2-YFP, HopH1-YFP, and HopAF1-YFP. Among them, HopAF1-YFP shows the most effective movement. More than 20% of transformation events result in the trafficking of HopAF1-YFP to two or more than two cell layers from the transformed cells (Figure 1B). In addition, we also choose HopAF1-YFP to confirm the method in determining the transformed plant cells by locating cells with the strongest HopAF1-YFP signals. We observed a similar pattern as HF-YFP. The transformed cells expressing ER-CFP always contain the strongest HopAF1-YFP signals within a cluster of cells containing YFP signals (Supplementary Figure 1A). For the majority of mobile effectors, around 20–30% of transformed cells exhibit the movement beyond initially transformed cells (Figure 1B). We then conducted immunoblot analysis to confirm the expression of full-length fusion proteins. Total proteins were extracted from *N. benthamiana* leaves transiently expressing the effector fusion proteins. The expression of the fusion proteins was detected using a GFP antibody. As shown in Supplementary Figure 3A, we detected a major band for most effectors at a higher molecular weight, suggesting that fluorescence signals detected in Figure 1A are emitted from full-length effector fusion proteins. It is noted that most fusion proteins migrated slower than expected (Supplementary Figure 3A; Supplementary Table 2). It is postulated that the higher molecular weight of the effector fusion proteins than expected might be due to post translational modifications within plants cells or unknow reasons. Together, the findings suggest that bacterial effectors are able to move beyond initially transformed cells.

### The Movement of Effectors Is Affected by Their Molecular Weights

**Figure 2:**
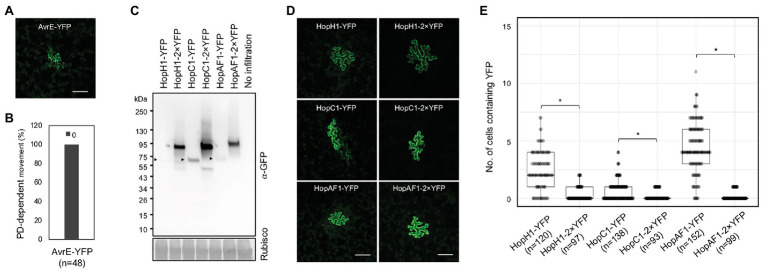
The movement of effectors is largely affected by their sizes. **(A)** Confocal image shows the expression of AvrE-YFP. Scale bars = 100 μm. **(B)** Quantitative data show that AvrE-YFP cannot move through PD. 0: no diffusion. The number of transformation events analyzed is indicated (*n*). **(C)** Detection of full-length effector fusion proteins. Effector-YFPs and effector-2×YFPs were transiently expressed in *N. benthamiana*. The samples were then subjected to immunoblot analysis using an anti-GFP antibody. Rubisco is served as a loading control. Arrow heads indicate the expression of effector-YFPs. **(D)** Confocal images show the diffusion of effector-YFP fusion proteins. Images were taken from epidermis of *N. benthamiana* transiently expressing different fusion proteins. Scale bars = 100 μm. **(E)** Quantitative data present the plasmodesmata (PD)-dependent movement of effector-YFP fusion proteins. Mann-Whitney *U* Test was used to analyze the data. The *p*-value is <0.0001 for HopH1-YFP vs. HopH1-2×YFP, <0.00094 for HopC1-YFP and HopC1-2×YFP, and <0.00001 for HopAF1-YFP and HopAF1-2×YFP (^*^). The number of transformation events analyzed is indicated (*n*).

Predicted molecular weights of most effector-YFP fusion proteins ranged between 50 and 80 kDa, whereas a few effectors like HopR1 and AvrE weight over 200 kDa (Supplementary Table 2). The majority of the mobile effector-YFPs shown in Figure 1 weights below 70 kDa, expect HopAA1-1-YFP (77.6 kDa; Supplementary Table 2). Among the tested effectors, AvrE-YFP does not move from transformed cells to the neighboring cells (Figures 2A,B). As *AvrE-YFP* encodes a protein with the molecular weight of ~222 kDa, the large molecule weight might impede the PD-dependent movement of the effector.

As we hypothesized that effectors move intercellularly through PD, we next examined whether the molecular weights of the mobile effectors affects their movement. We thus constructed HopH1, HopC1, and HopAF1 with two tandem repeats of YFP, yielding HopH1-2×YFP, HopC1-2×YFP, and HopAF1-2×YFP. We first determined the molecular weights of the fusion proteins by transiently expressing them in *N. benthamiana* leaves using an Agrobacterium-mediated approach. We then detected the expression of the fusion proteins using a GFP antibody. Compared to 1×YFP fusion, 2×YFP fusion of the effectors increases the molecular weight by ~26 kDa (Figure 2C). To investigate the PD-dependent movement of 2×YFP fusion proteins, we determined the movement of HopH1-2×YFP, HopC1-2×YFP, and HopAF1-2×YFP in *N. benthamiana* leaves as mentioned above. Compared to the diffusion of 1×YFP fusion proteins, the movement of HopH1-2×YFP, HopC1-2×YFP, and HopAF1-2×YFP beyond initially transformed cells is drastically reduced (Figures 2D,E). Together, the findings support that the bacterial effectors move between plant cells through PD.

### The Expression of PDLP5 and PDLP7 Suppresses the PD-Dependent Movement of HopAF1

**Figure 3:**
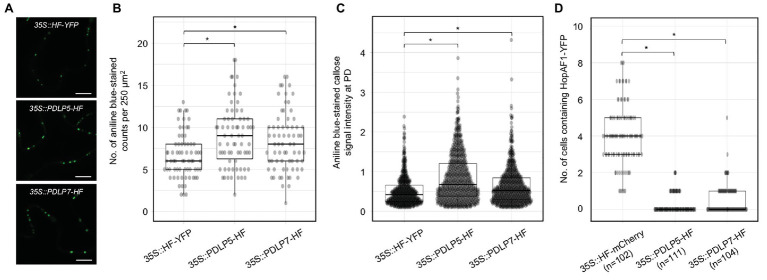
Expression of PDLP5 and PDLP7 suppresses the PD-dependent movement of HopAF1. **(A)** Expression of plasmodesmata-located proteins (PDLPs) affects callose accumulation at PD. *N. benthamiana* leaves were infiltrated with Agrobacteria harboring *35S::HF-YFP*, *35S::PDLP5-HF*, and *35S::PDLP7-HF*. Infiltrated leaves were stained with 0.01% aniline blue and imaged with confocal microscopy. Scale bars = 10 μm. **(B)** Quantitative data present the number of aniline blue-stained callose at PD per 250 μm^2^ of *N. benthamiana* epidermis. Mann-Whitney *U* Test was used to analyze the data. The *p*-value is <0.05 (^*^). **(C)** Quantitative data present the accumulation of callose at PD. Mann-Whitney *U* Test was used to analyze the data. The *p*-value is <0.0001 (^*^). **(D)** Quantitative data present the PD-dependent movement of HopAF1-YFP when co-expressed with *35S::HF-mCherry*, *35S::PDLP5-HF*, and *35S::PDLP7-HF*. Mann-Whitney *U* Test was used to analyze the data. The *p*-value is <0.00001 for both *35S::PDLP5-HF* and *35S::PDLP7-HF* (^*^). The number of transformation events analyzed is indicated (*n*).

Altered expression of PDLPs has been shown to impact the PD function. To further support that the intercellular movement of effectors depends on PD, we investigated whether the expression of PDLP affects the movement of bacterial effectors. PDLP5 has been shown to affect callose deposition at PD and alter the movements of GFP molecules between cells in *Arabidopsis*; however, it’s unknown whether PDLPs regulates callose accumulation at PD when transiently expressed in *N. benthamiana*. To this end, we detected callose accumulation at PD in *N. benthamiana* after PDLP5 or PDLP7 was transiently expressed. PDLP5 and PDLP7 were selected due to their role in bacterial immunity (Lee et al., 2011; Aung et al., 2020). We first detected the expression of HF-YFP (mock), PDLP5-HF, and PDLP7-HF using immunoblot analysis (Supplementary Figure 3B). The leaf transiently expressing the fusion proteins was stained with aniline blue to detect callose accumulated at PD. Similar to *Arabidopsis* transgenic plants overexpressing PDLP5 (Lee et al., 2011), transient expression of *Arabidopsis* PDLP5 is sufficient to increase callose accumulation at PD compared to that of mock treatment (Figures 3A–C). While PDLP5 has been previously shown to regulate callose homeostasis, whether PDLP7 has similar roles in callose accumulation has not been determined. Here, we demonstrated that transient expression of *Arabidopsis* PDLP7 also leads to higher accumulation of callose at PD in *N. benthamiana* (Figures 3A–C). Together, the findings showed that transient overexpression of the PDLP5 and PDLP7 could increase the callose accumulation at PD in *N. benthamiana*.

Callose accumulation at PD is negatively associated with PD-dependent movement of molecules between plant cells (De Storme and Geelen, 2014; Amsbury et al., 2017; Wu et al., 2018). To further support that bacterial effectors move through PD, we investigated whether PDLP-mediated PD closure would suppress the movement of a bacterial effector HopAF1. Among the mobile effectors, HopAF1 was chosen in this assay because of its highest PD-dependent movement in plants (Figure 1). Relatively lower inoculum (A_600_ 2 × 10^−4^) of Agrobacteria harboring *35S::HopAF1-YFP* was mixed with a higher inoculum (A_600_ 0.1) of Agrobacteria harboring *35S::HF-mCherry* (mock), *35S::PDLP5-HF*, or *35S::PDLP7-HF* and infiltrated into *N. benthamiana* leaves. The expression of the fusion proteins was determined using immunoblot analysis (Supplementary Figure 3C). The movement of HopAF1-YFP was determined 2 days after the Agrobacterium infiltration using confocal microscopy. As shown in Figure 3D, the expression of PDLP5 and PDLP7 drastically reduced the intercellular movement of HopAF1-YFP beyond the transformed cells. Together, the findings suggest that the expression of PDLP5 and PDLP7 affects the PD-dependent movement of a bacterial effector.

### flg22 Inhibits the Movement of a Bacterial Effector

**Figure 4:**
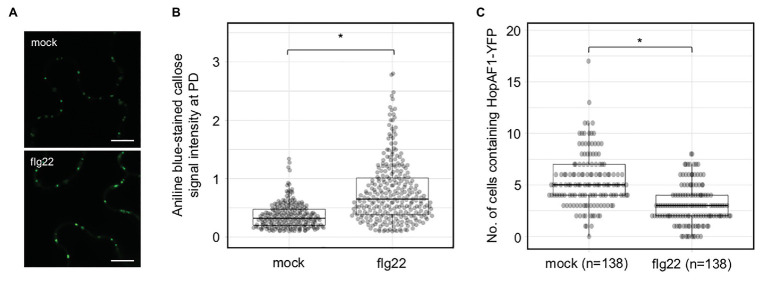
Pattern-triggered immunity (PTI)-induced callose accumulation at PD reduces the movement of effectors. **(A)** Flagellin (flg22) induces callose accumulation at PD. *Nicotiana benthamiana* leaves were infiltrated with 0.1 μM of flg22 or ddH_2_O (mock). Infiltrated leaves were stained with 0.01% aniline blue and imaged with confocal microscopy. Scale bars = 10 μm. **(B)** Quantitative data present the accumulation of callose at PD. Mann-Whitney *U* Test was used to analyze the data. The *p*-value is <0.00001 (^*^). **(C)** flg22 treatment suppresses the PD-dependent movement of HopAF1. *Nicotiana benthamiana* leaves were pretreated with 0.1 μM of flg22 or ddH_2_O (mock) for 24 h. Agrobacteria harboring *35S::HopAF1-YFP* were later infiltrated into the pretreated leaves. The PD-dependent movement of HopAF1-YFP was examined 48 h post Agrobacterium infiltration using confocal microscopy. The data shown here were collected from four biological repeats. Mann-Whitney *U* Test was used to analyze the data. The *p*-value is <0.00001 (^*^). The number of transformation events analyzed is indicated (*n*).

In addition to PDLP expression, flg22 has been also reported to induce callose deposition at PD and reduce the PD-dependent molecular fluxes between cells in *Arabidopsis* (Faulkner et al., 2013; Xu et al., 2017). To determine the effect of flg22 on callose accumulation at PD in *N. benthamiana*, we treated a fully expended leaf of *N. benthamiana* with 0.1 μM flg22. Callose deposition at PD was examined 24 h after the infiltration. flg22-treated leaf, compared to mock-treated leaf (infiltrated with ddH_2_O), exhibits higher accumulation of callose at PD (Figures 4A,B). We next determined whether flg22 treatment suppresses the movement of HopAF1 using an Agrobacterium-mediated protein movement assay mentioned above. The expression of HopAF1-YFP was detected using immunoblot analysis (Supplementary Figure 3D). In line with the callose accumulation at PD, flg22-treatment inhibits the PD-dependent movement of HopAF1 (Figure 4C).

### PD-Dependent Movement of the PM-Associated HopAF1

**Figure 5:**
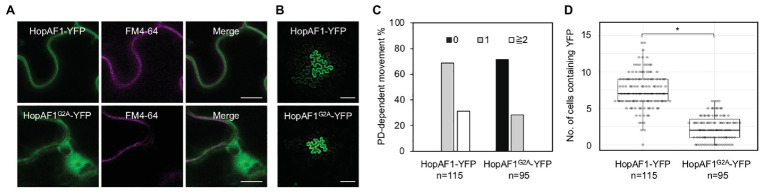
The plasma membrane (PM) association of HopAF1 does not inhibit the PD-dependent movement. **(A)** *Nicotiana benthamiana* leaves transiently expressing HopAF1-YFP or HopAF1^G2A^-YFP were stained with FM4–64 to label the PM. Confocal images show the PM localization of HopAF1-YFP and the nucleocytoplasmic localization of HopAF1^G2A^-YFP. Scale bars = 10 μm. **(B)** Confocal images show the PD-dependent movement of HopAF1-YFP or HopAF1^G2A^-YFP determined by an Agrobacterium-mediated protein movement assay. Images were taken from the epidermis of *N. benthamiana* leaves. The transformed plant cell exhibits strong YFP signals. The movement of the fusion proteins is determined by the detection of YFP signals in cells surrounding the transformed cell. Scale bars = 100 μm. **(C)** Quantitative data show the percentage of transformation events resulting in no diffusion (0), one cell layer diffusion (1), and two or more than two cell layers diffusion (≧2). The data shown here are pooled from at least three biological replicates. The number of transformation events analyzed is indicated (*n*). **(D)** Quantitative data present the numbers of surrounding plant cells to the transformed cells containing YFP signals. Mann-Whitney *U* Test was used to analyze the data. The *p*-value is <0.00001 (^*^). The number of transformation events analyzed is indicated (*n*).

Among the mobile effectors, HopAF1 and HopA1 are detected on the PM in plant cells (Supplementary Figure 2). The PM localization of HopA1 and HopAF1 has been previously reported (Toruno Calero, 2014; Washington et al., 2016). As HopAF1 contains a putative *N*-myristolation site (G2), we postulated that the PM association of HopAF1 is mediated through the protein lipidation. To determine the PM association of HopAF1 in an *N*-myristolation-dependent manner, we constructed a G2A mutant of HopAF1-YFP (HopAF1^G2A^-YFP). Using the Agrobacterium-mediated transient expression approach, HopAF1-YFP and HopAF1^G2A^-YFP were expressed in *N. benthamiana*. The expression of HopAF1-YFP and HopAF1^G2A^-YFP was detected using a GFP antibody (Supplementary Figure 3A). To stain the PM, Agrobacterium-infected leaves were infiltrated with FM4-64 dye. HopAF1-YFP overlapped with the FM4-64 stained PM in *N. benthamiana*, confirming the PM association of HopAF1-YFP. As predicted, HopAF1^G2A^-YFP was not associated with the PM. Instead, HopAF1^G2A^-YFP was detected in the cytosol and nucleus (Figure 5A). The findings suggest that the PM association of HopAF1-YFP is mediated through the *N*-myristoylation at the N-terminal Glycine (G2).

We next determined the PD-dependent movement of HopAF1^G2A^-YFP using the Agrobacterium-mediated protein movement assay in *N. benthamiana*. It was assumed that the PM association of molecules might negatively impact the PD-dependent movement. Surprisingly, the nucleocytoplasmic localized HopAF1^G2A^-YFP is not as mobile as the PM associated HopAF1-YFP. Only around 30% of the transformation events of HopAF1^G2A^-YFP led to the PD-dependent movement compared to HopAF1-YFP, in which all transformation events led to the PD-dependent movement (Figures 5B–D). The findings indicate that the PM association of HopAF1 does not negatively affect the PD-dependent movement of the protein.

## Discussion

The PD-dependent movement of fungal effectors (Khang et al., 2010) and an oomycete effector (Khang et al., 2010; Cao et al., 2018; Tomczynska et al., 2020) have been reported; however, it’s unclear whether bacterial effectors move between plant cells or not. Empirical evidence from this work showed that at least 16 *Pst* DC3000 effectors move between plant cells through PD. We established that the movement of the effectors is dependent on PD from the following findings: (1) the effector-YFP fusion proteins can move from the transformed cells to the adjoining plant cells (Figure 1), (2) the movement of the effectors is largely dependent on their molecular weights (Figure 2), and (3) PDLP5-, PDLP7-, and flg22-induced callose accumulation at PD inhibits the movement of a bacterial effector HopAF1 (Figures 3, 4).

Although the movement of 16 effectors is reported here, it’s plausible that more *Pst* DC3000 effectors are able to move between plants cells. The following reasons might account for the underestimation of the PD-dependent movement of bacterial effectors: (1) the YFP fusion of effectors increases their molecular weights and could suppress their PD-dependent movement, (2) transiently overexpressing individual effector induces cell death in *N. benthamiana* thus preventing the visualization of the effectors, and (3) the expression level of some effectors is under the detectable threshold using confocal microscopy.

It is well established that the molecular weight of proteins affects their movement between plant cells through PD (Kim et al., 2005; Aung et al., 2020). In both *Arabidopsis* and *N. benthamiana*, the movement of 2×YFP and 3×YFP is greatly inhibited. Among the effectors we investigated, we did not observe the movement of AvrE-YFP (Figures 2A,B). The expression of DEX-His-AvrE was detected at ~250 kDa in *Arabidopsis* (Xin et al., 2015). Also, the tandem fusion of YFP to HopH1-YFP, HopC1-YFP, and HopAF1-YFP (yielding HopH1-2×YFP, HopC1-2×YFP, and HopAF1-2×YFP) drastically suppresses the PD-dependent movement (Figures 2D,E). The addition of another YFP increases the molecular weight of the fusion proteins by ~26 kDa (Figure 2C). It is also possible that the tandem fusion of YFP affects the tertiary structure of the fusion proteins, impeding the PD-dependent movement.

Many mobile effectors were detected both in the nucleus and cytoplasm (Supplementary Figure 2); however, we observed the PD-dependent movement of the PM-localized effectors, HopAF1 and HopA1. Interestingly, the PM-associated HopAF1 is the most mobile effector among the 16 effectors reported here (Figure 1B). A few *Pst* DC3000 effectors have been reported to associate with the PM of plant cells (Shan et al., 2000; Göhre et al., 2008; Xin et al., 2015; Washington et al., 2016; Aung et al., 2020), whereas none of *Pst* DC3000 effectors contains putative transmembrane domains. It was previous reported that mutations in putative sites for myristoylation (G2) and palmitoylation (C4) of HopAF1 (HopAF1^G2AC4S^-cerulean-HA) abolishes the PM localization (Washington et al., 2016). Similar to HopO1-1 and AvrPto1 (Shan et al., 2000; Aung et al., 2020), the G2A mutation is sufficient to disrupt the PM association of HopAF1 (Figure 5A). Interestingly, the nucleocytoplasmic localized HopAF1^G2A^-YFP is not as mobile as the wild-type HopAF1-YFP (Figures 5B–D). It is worth pursuing whether the PM association of effectors facilitates the PD-dependent movement of molecules along the PM lining the PD channel. Together, the findings suggest that the membrane association of effectors does not inhibit the PD-dependent movement. It is unclear whether mitochondrial, chloroplast, or the ER association of effectors affects the PD-dependent movement.

In *Arabidopsis*, the expression of PDLP5 is positively correlated with the accumulation of callose at PD (Lee et al., 2011). Here, we reported that the transient overexpression of *Arabidopsis* PDLP5 in *N. benthamiana* increases the accumulation of callose at PD (Figures 3A,B). The finding is supported by a recent report that the transient overexpression of PDLP5 suppresses the PD-dependent movement of mCherry (Wang et al., 2020). Similar to PDLP5, the transient expression of *Arabidopsis* PDLP7 also increases callose accumulation at PD in *N. benthamiana* (Figures 3A,B). Among different PDLP members, only the expression of PDLP5 transcripts and proteins is upregulated by bacterial infections and a defense hormone SA treatment (Lee et al., 2011). PDLP7 proteins are destabilized by *P. syringae* infection in a bacterial effector HopO1-1-dependent manner (Aung et al., 2020). Given that HopO1-1 physically associates with and destabilizes PDLP5 and PDLP7, the effector might target the PDLPs to suppress plasmodesmal immunity. The targeting of the PDLPs might play critical role in facilitating the PD-dependent movement of bacterial effectors from the infected cells to the adjoining non-infected cells through PD. In line with the notion, we observed that the transient overexpression of PDLP5 and PDLP7 significantly suppresses the PD-dependent movement of a highly mobile bacterial effector HopAF1.

Pathogen-associated molecular pattern-triggered callose accumulation at PD suggests that the plasmodesmal closure is a part of pattern-triggered immunity (PTI). PTI is considered the first line of plant immune responses during microbial infection (Boller and He, 2009). It is plausible that plants induce the plasmodesmal closure to limit the spread of microbial molecules from the infected cells to the surrounding plant cells. In line with the statement, flg22 treatment suppresses the PD-dependent movement of a bacterial effector HopAF1 (Figure 4C). It is postulated that the PTI-triggered callose accumulation at PD generally suppresses the PD-dependent movement of most effectors. Recent report showed that the expression of a bacterial effector HopO1-1 facilitates the PD-dependent movement of YFP molecules (Aung et al., 2020). As HopO1-1 targets and destabilizes PDLP5 and PDLP7, it is highly plausible that HopO1-1 functions to overcome the plasmodesmal immunity. We thus hypothesize that HopO1-1 might facilitate the PD-dependent movement of bacterial effectors to the surrounding plant cells. The hypothesis is supported by a recent report that the PD-dependent cell-to-cell movement of *F. oxysporum* effector Avr2-GFP requires Six5 (Cao et al., 2018). Further studies will reveal the role of HopO1-1 in modulating the PD-dependent movement of bacterial effectors.

Although the function of many *Pst* DC3000 effectors has been predicted according to their amino acid sequences, only a handful of them has been confirmed their activities *in planta* (Supplementary Table 2). As effector proteins are believed to be involved in suppressing plant immunity to benefit the microbes, the mobile effectors might play crucial roles in inhibiting non-cell-autonomous plant immunity. Successful suppression of both cell-autonomous and non-cell-autonomous plant immunity might be critical for pathogenic microbes to colonize and spread from the initial infection sites. Understanding the function of mobile effectors will allow us to better understand how pathogenic microbes regulate cellular processes in infected plant cells and the surrounding plant cells. This report also demonstrates that an Agrobacterium-mediated protein movement assay using *N. benthamiana* is a powerful experimental system to determine the PD-dependent movement of microbial effectors. The system has great potential in directly visualizing how the mobile effectors affect plant immune responses. Identification and characterization of robust plant immune response markers will allow us to investigate the functions of mobile effectors in modulating cell-autonomous and non-cell-autonomous immune responses *in planta*.

## Data Availability Statement

The original contributions presented in the study are included in the article/Supplementary Material; further inquiries can be directed to the corresponding author.

## Author Contributions

KA designed the research, conducted the experiments shown in Figures 3D, 5A; Supplementary Figures 2, 3,and wrote the manuscript with input from all authors. ZL performed the experiments shown in Figures 1, 2A,B, 5B–D, and Supplementary Figures 1, 3A. HV conducted the experiments shown in Figures 3A–C, 4. YC conducted the experiments shown in Figures 2D,E. S-LL contributed to Figure 2C and Supplementary Figure 3. All authors contributed to the article and approved the submitted version.

### Conflict of Interest

The authors declare that the research was conducted in the absence of any commercial or financial relationships that could be construed as a potential conflict of interest.

## Footnotes

^1^https://huygens.science.uva.nl/PlotsOfData/

^2^https://www.socscistatistics.com/tests/mannwhitney/default2.aspx
