# Supplementary material for: Plasmodesmata-Dependent Intercellular Movement of Bacterial Effectors
Source: Front Plant Sci. 2021 Mar 22;12:640277. doi: 10.3389/fpls.2021.640277 (PMC8095247; doi:10.3389/fpls.2021.640277)
Supplement: Supplementary file 1 [file Table_1.pdf]

Supplemental Table 1. Primers used for cloning in this study.

Primer	Sequence	For cloning
HopH1-attB1	ggggacaagtttgtacaaaaaagcaggcttcatgatcactccgtctcgatatc	HopH1-2×YFP
HopH1-YFP-F	gtacagggcacatcaaatggtgagcaagggcg	HopH1-2×YFP
HopH1-YFP-R	cgcccttgctcaccatttgatgtgccctgtac	HopH1-2×YFP
EYFP-attB2	ggggaccactttgtacaagaaagctgggtcttacttgtacagctcgtcc	HopH1-2×YFP
HopC1-attB1	ggggacaagtttgtacaaaaaagcaggcttcatgacaatcgtgtctggac	HopC1-2×YFP
HopC1-YFP-F	gtattcgcttcaaaaatacacatggtgagcaagggc	HopC1-2×YFP
HopC1-YFP-R	gcccttgctcaccatgtgtatttttgaagcgaatac	HopC1-2×YFP
EYFP-attB2	ggggaccactttgtacaagaaagctgggtcttacttgtacagctcgtcc	HopC1-2×YFP
HopAF1-attB1	ggggacaagtttgtacaaaaaagcaggcttcatggggctatgtatttcaaaac	HopAF1-2×YFP
HopAF1-YFP-F	catctggtcgcacaaatggtgagcaagggcg	HopAF1-2×YFP
HopAF1-YFP-R	cgcccttgctcaccatttgtgcgaccagatg	HopAF1-2×YFP
EYFP-attB2	ggggaccactttgtacaagaaagctgggtcttacttgtacagctcgtcc	HopAF1-2×YFP
mCherry-attB1	ggggacaagtttgtacaaaaaagcaggcttcatggtgagcaagggcg	HF-mCherry
mCherry-attB2	ggggaccactttgtacaagaaagctgggtcttacttgtacagctcgtcc	HF-mCherry
HopAF1^{G2A}-attB1	ggggacaagtttgtacaaaaaagcaggcttcatggcgctatgtatttcaaaacac	HopAF1^{G2A}-YFP
HopAF1-attB2	ggggaccactttgtacaagaaagctgggtcttgtgcgaccagatgttttatg	HopAF1^{G2A}-YFP
