# Supplementary material for: Plasmodesmata-Dependent Intercellular Movement of Bacterial Effectors
Source: Front Plant Sci. 2021 Mar 22;12:640277. doi: 10.3389/fpls.2021.640277 (PMC8095247; doi:10.3389/fpls.2021.640277)
Supplement: Supplementary file 2 [file Table_2.pdf]

Supplemental Table 2. *Pst* DC3000 effector-YFP fusion proteins.

Fusion protein	PD-dependent movement	Cell death induction	Predicted molecular weight (kDa)	Putative or confirmed (*) functions	References
HopK1-YFP	Y		63.4		
HopY1-YFP	Y		57.9		
HopF2-YFP	Y		49.3	ADP-ribosyltransferase*	Wang et al., 2010
HopU1-YFP	Y		56.9	ADP-ribosyltransferase*	Fu et al., 2007
HopH1-YFP	Y	Y	51.2	Peptidase	
HopC1-YFP	Y		55.8	Cysteine protease	
HopD1-YFP	n.d.	Y	100.9		
HopQ1-YFP	n.d.	Y	74.7	Nucleoside hydrolase	Li et al., 2013
HopR1-YFP	n.d.	Y	236.4		
HopAM1-1-YFP	n.d.	Y	57.3		
HopN1-YFP	Y	Y	65.4	Cysteine protease	López-Solanilla et al., 2004
HopAA1-1-YFP	Y		77.6	GTPase-activating protein	Munkvold et al., 2009
AvrE1-YFP	N	Y	222		
HopAF1-YFP	Y		57.8	Demidase	Washington et al., 2016
HopP1-YFP	Y		59.3	Lytic transglycosylases	Kvitko et al., 2007
HopAB2-YFP	Y		86.3	E3 ubiquitin-protein transferase*	Abramovitch et al., 2006
AvrPto1-YFP	n.d.	Y	44.3		
HopE1-YFP	Y		50.9		
HopAA1-2-YFP	Y		78	GTPase-activating protein	Munkvold et al., 2009
HopV1-YFP	n.d.		69.1		
HopAO1-YFP	Y	Y	78.3	Tyrosine phosphatase*	Macho et al., 2014
HopG1-YFP	n.d.		80.7		
HopI1-YFP	Y		79.5		
HopA1-YFP	Y	Y	68.9	Phosphothreonine lysase	Toruno Calero, 2014

HopX1-YFP	Y		68.3	Cystine protease	
HopAM1-2-YFP	n.d.	N	57.3		
HopO1-1-YFP	n.d.		57.4	ADP-ribosyltransferase	Aung et al., 2020
HopT1-1-YFP	n.d.	Y	67.1		
HopB1-YFP	n.d.		76.3	Protease	Li et al., 2016

Y: Yes, N: No, n.d.: Not determined.

REFERENCES:

Abramovitch, R., Janjusevic, R., Stebbins, C.E., and Martin, G.B. 2006. Type III effector AvrPtoB requires intrinsic E3 ubiquitin ligse activity to suppress plant cell death and immunity. Proc. Natl. Acad. Sci. USA **108:** 2851-2856.

Fu, Z.Q., Guo, M., Jeong, B.R., Tian, F., Elthon, T.E., Cerny, R.L., Staiger, D., and Alfano, J.R. 2007. A type III effector ADP-ribosylates RNA binding proteins and quell plant immunity. Science **447,** 284-288.

Macho, A.P., Schwessinger, B., Ntoukakis, V., Brutus, A., Segonzac, C., Roy, S., Kadota, Y., Oh, M., Sklenar, J., Derbyshire, P., Lozano-Durán, R., Malinovsky, F.G., Monaghan, J., Menke, F.L., Huber, S.C., He, S.Y., and Zipfel, C. 2014. A bacterial tyrosine phosphatase inhibits plant pattern recognition receptor activation. Science **6178:** 1509-1512.

Munkvold, K.R., Russel, A.B., Kvitko, B.H., and Collmer, A. 2009. Pseudomonas syringae pv. tomato DC3000 type III effector HopAA1-1 functions redundantly with chlorosis-promoting factor PSPTO4723 to produce bacterial speck lesions in host tomato. Mol. Plant Microbe In. **22,** 1341-1355.

Kvitko, B.H., Ramos, A.R., Morello, J.E., Oh, H., and Collmer, A. 2007. Identification of harpins in Pseudomonas syringae pv. tomato DC3000 which are functionally similar to HrpK1 in promoting translocation of type III secretion system effectors. J. Bacteriol. **189,** 8059-8072.

Li, L., Kim, P., Yu, L., Cai, G., Chen, S., Alfano , J.R., and Zhou, J. 2016. Activation-dependent destruction of a co-receptor by a *Pseudomonas syringae* effector dampens plant immunity. Cell Host Microbe **20,** 504-514.

Li, W., Chiang, Y., and Coaker, G. 2013. The HopQ1 effecctor's nucleoside hydrolase-domain is required for bacterial virulance in Arabidopsis and Tomato, but not host recognition in tobacco. Plos One https://doi.org/10.1371/journal.pone.0059684.

López-Solanilla, E., Bronstein, P.A., Schneider A.R., and Collmer, A. 2004. HopPtoN is a *Pseudomonas syringae* Hrp (type III secretion system) cysteine protease effector that suppresses pathogen-induced necrosis associated with both compatible and incompatible plant interactions. Mol. Microbiol. **54,** 353-365.

Toruno Calero, T.Y. 2014. *Pseudomonas syringae* type III effectors: Targets and roles in plant immunity. ETD collection for University of Nebraska – Lincoln. AAI3667423.

Wang, Y., Li, J., Hou, S., Wang, X., Li, Y., Ren, D., Chen, S., Tang, X., Zhou, J. 2010. A Pseudomonas syringae ADP-Ribosyltransferase inhibit Arabidopsis Mitogen-Activated Protein kinase kinases. Plant Cell **22:** 2033-2044.
