# Supplementary figures and images for: Plasmodesmata-Dependent Intercellular Movement of Bacterial Effectors
Source: Front Plant Sci. 2021 Mar 22;12:640277. doi: 10.3389/fpls.2021.640277 (PMC8095247; doi:10.3389/fpls.2021.640277)

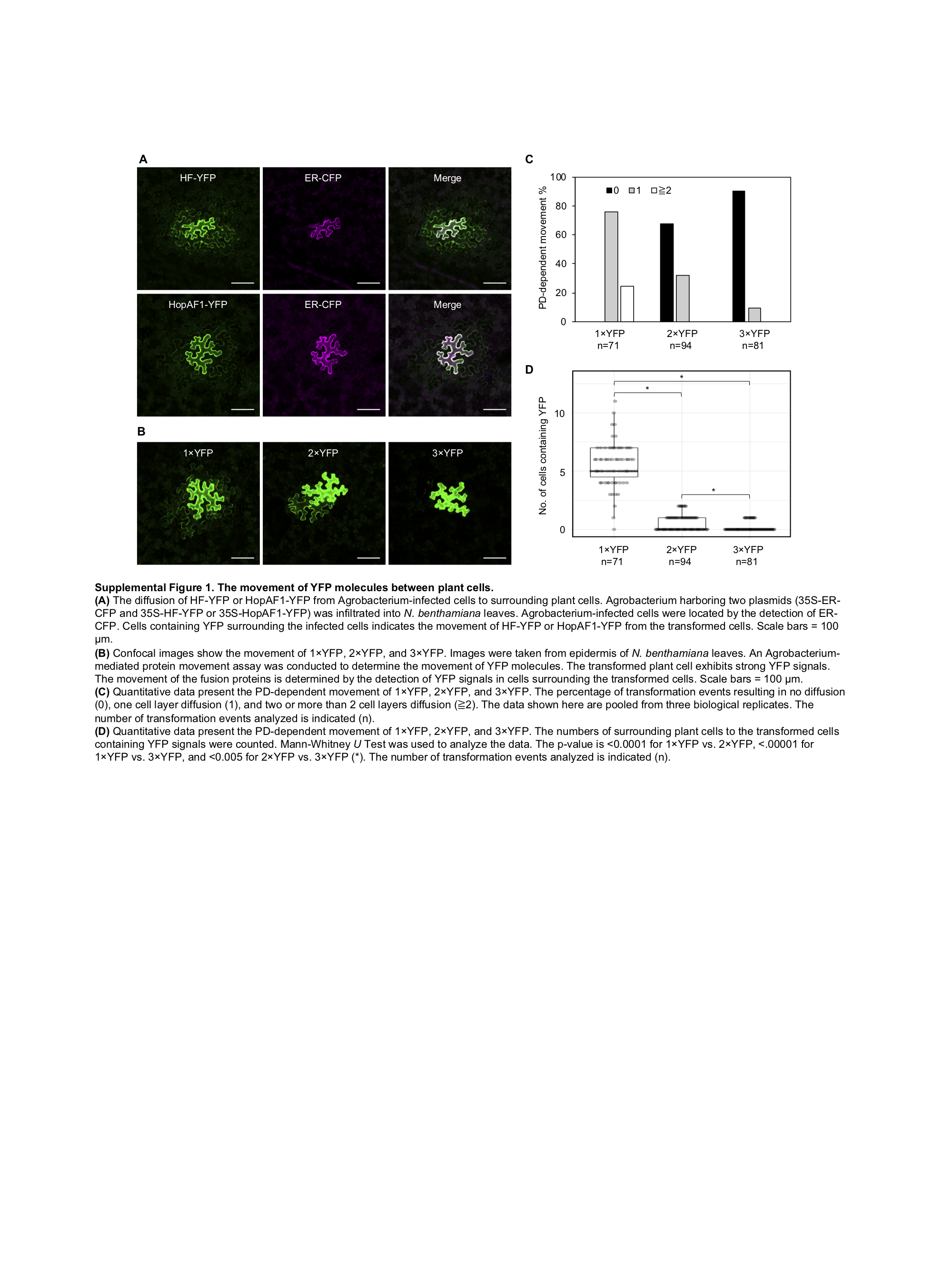

Supplement: Supplementary file 3 [file Image_1.tiff]

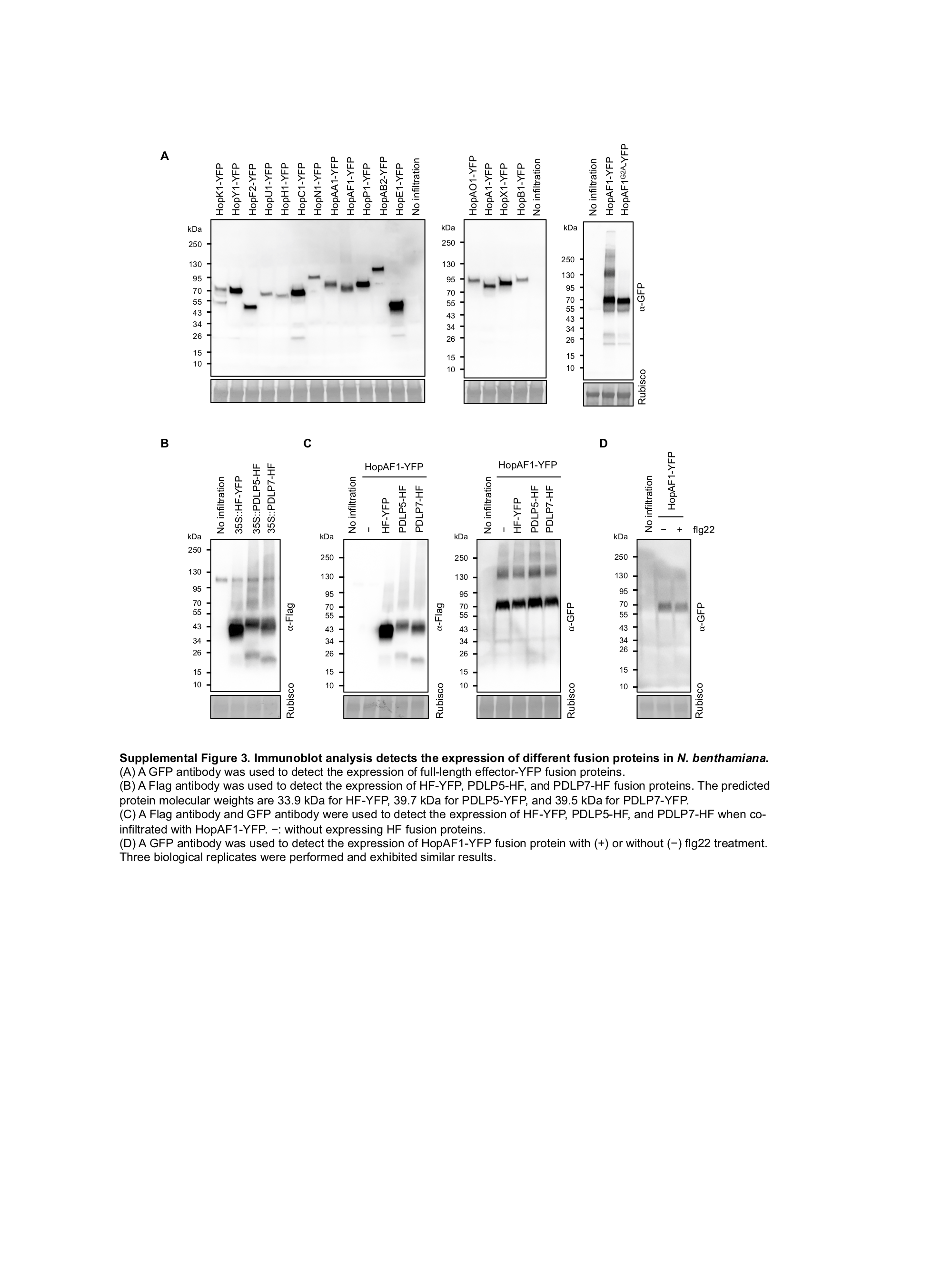

Supplement: Supplementary file 5 [file Image_3.tiff]
