# Supplementary material for: Plasmodesmata-Dependent Intercellular Movement of Bacterial Effectors
Source: Front Plant Sci. 2021 Mar 22;12:640277. doi: 10.3389/fpls.2021.640277 (PMC8095247; doi:10.3389/fpls.2021.640277)
Supplement: Supplementary file 4 [file Image_2.pdf]

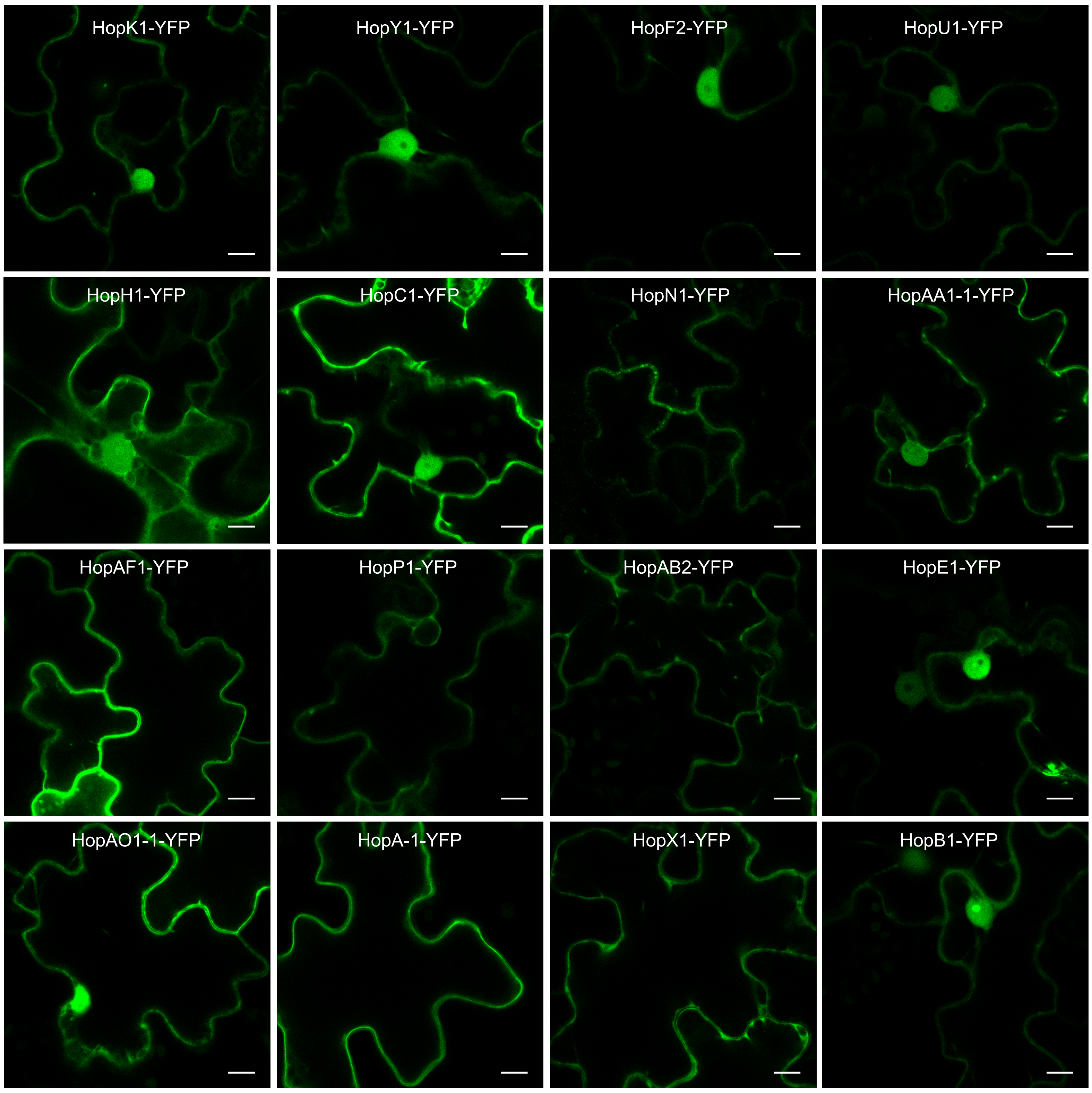

Supplemental Figure 2. Transient expression of bacterial effector proteins in *N. benthamiana*.
Expression and subcellular localization of bacterial effector proteins. Images were taken with confocal microscopy. Scale bars = 10 μm.
